# Does the treatment concept of advanced gastric cancer require innovation?: Two cases of advanced gastric cancer with distinctively different outcomes

**DOI:** 10.1097/MD.0000000000048628

**Published:** 2026-05-08

**Authors:** Meifeng Zhang, Xue Ding, Li Dai, Hongbin Qi, Siqi Luo, Xiangren Jin, Qian Wang

**Affiliations:** a Department of Outpatient Clinic, Affiliated Hospital of Guizhou Medical University, Guiyang, Guizhou Province, China; b Department of Gastrointestinal Surgery, Affiliated Hospital of Guizhou Medical University, Guiyang, Guizhou Province, China.

**Keywords:** advanced gastric cancer, neoadjuvant chemotherapy

## Abstract

**Rationale:**

With the continuous progress of science and technology, remarkable achievements have been made in the treatment of gastric cancer (GC). In China, the vast majority of GC patients are diagnosed with advanced GC at the time of initial detection. Given its high incidence and mortality rates, GC poses a serious threat to the health of the Chinese population. The selection of appropriate treatment strategies for patients with advanced GC remains a controversial and challenging issue in clinical practice.

**Patient concerns:**

Two patients diagnosed with advanced GC experienced drastically different prognoses due to the adoption of disparate treatment modalities.

**Diagnoses:**

One patient, at stage IIIA, another patient at stage IIB.

**Interventions:**

The stage IIIA patient received neoadjuvant chemotherapy followed by surgical resection, and the stage IIB patient underwent direct surgical resection.

**Outcomes:**

The patient who received neoadjuvant therapy and surgery has remained free of recurrence so far. In contrast, the direct surgery one unfortunately succumbed to tumor recurrence 1 year after the operation.

**Lessons:**

These 2 patients with the same diagnosis of advanced GC presented strikingly different outcomes, which prompted us to deeply contemplate the optimal treatment approaches for this disease. This paper aims to provide valuable references for the treatment selection of advanced GC.

## 1. Introduction

Gastric cancer is one of the most prevalent tumors in the digestive system. Globally, it ranks as the 5th most common malignant tumor and is the 4th leading cause of cancer-related death.^[1]^ Southeast Asia is currently recognized as the region with the highest incidence and mortality rates of GC. In China, in particular, its incidence rate is the second highest among all malignant tumors.^[2,3]^ Due to the nonspecific symptoms of early stage GC and the limited use of endoscopy as a routine screening method, approximately 70.8% of Chinese GC patients are diagnosed with locally advanced disease at their first medical visit. Radical surgery remains the main curative treatment for GC. However, even after undergoing radical resection, 40% to 60% of patients may experience tumor recurrence or metastasis, leading to a 5-year overall survival rate of <30% and a generally poor prognosis.^[4]^

At the dawn of the 21st century, the treatment of GC has gradually shifted from a single surgical approach to a comprehensive treatment model centered around surgery. Although radical surgical treatment is still the most effective option for advanced GC, in recent years, the clinical importance of perioperative treatment for locally advanced gastric cancer (LAGC) has drawn increasing attention, thanks to the continuous exploration and application of various perioperative treatment methods. Different from the previous single-surgical treatment mode, neoadjuvant chemotherapy has shown promising therapeutic effects. Here, we report 2 cases of patients who received either single surgical resection or neoadjuvant chemotherapy combined with surgery. These 2 patients, with different treatment modalities, achieved significantly different therapeutic outcomes.

## 2. Case 1

**Figure 1.**
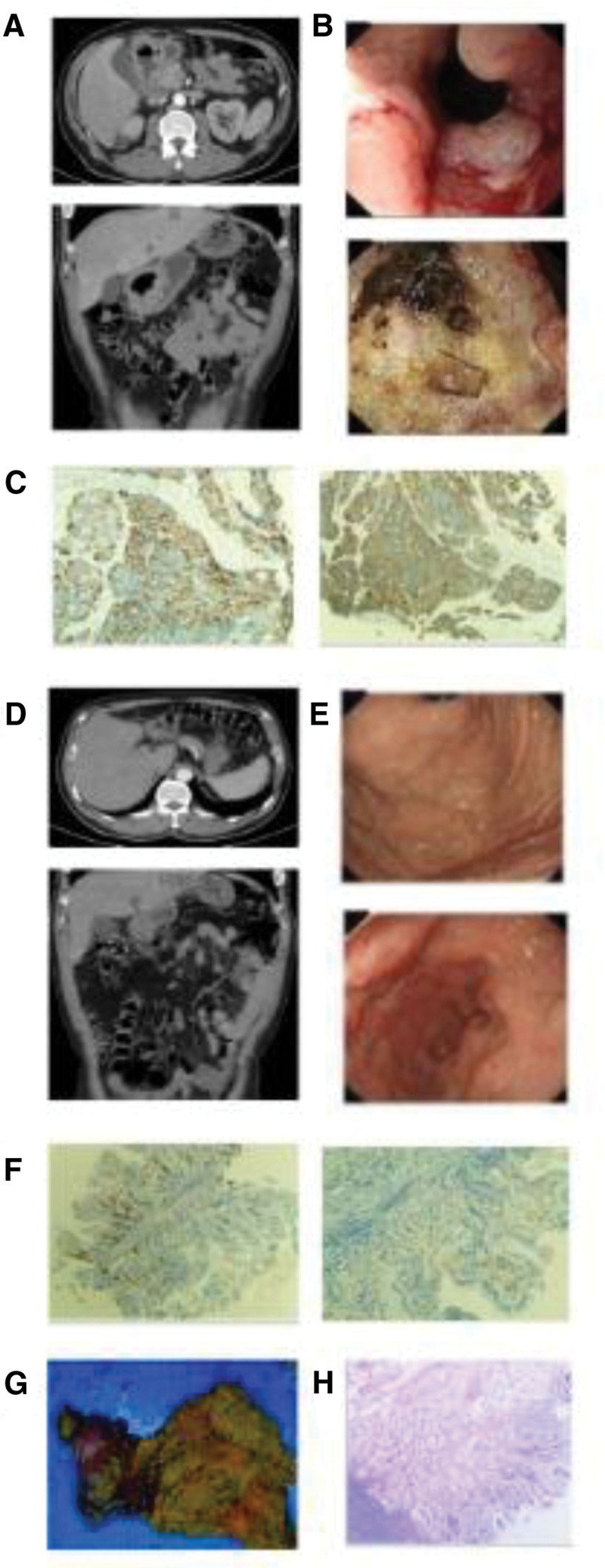
(A) Computed tomography before the neoadjuvant chemotherapy; (B) gastroscopy before the neoadjuvant chemotherapy; (C) pathological examination before the neoadjuvant chemotherapy; (D) computed tomography after the neoadjuvant chemotherapy. (E) Gastroscopy after the neoadjuvant chemotherapy; (F) pathological examination after the neoadjuvant chemotherapy; (G) postoperative specimen; (H) postoperative pathological examination.

In March 2020, a 57-year-old male patient was hospitalized with a chief complaint of upper abdominal distension, discomfort, accompanied by acid reflux and vomiting that had persisted for over 2 months. Hematological examination results showed moderate anemia, while tumor biomarker levels remained within normal ranges. Computed tomography (CT) scans demonstrated uneven thickening of the gastric antrum wall, with local ulcer formation and enlarged surrounding lymph nodes (Fig. 1A). Gastroscopy revealed a large ulcerative lesion spanning the gastric antrum, pyloric canal, and duodenal bulb, measuring approximately 7 × 4 cm (Fig. 1B). Pathological analysis confirmed the diagnosis of poorly differentiated adenocarcinoma of the gastric antrum (Fig. 1C). The clinical stage was determined to be cT4aN1M0, corresponding to stage IIIA.

Based on current treatment guidelines, for patients with stage IIIA non-esophagogastric junction tumors, options include selective surgical treatment or neoadjuvant chemotherapy followed by surgery. At that time, the G208 clinical trial was recruiting participants. After comprehensive discussion with the medical team, the patient expressed concerns about being allocated to the SOX treatment group and ultimately decided not to enroll in the clinical trial. Instead, he chose a privately funded neoadjuvant chemotherapy treatment regimen that combined immunotherapy, chemotherapy, and targeted therapy. This regimen consisted of Camrelizumab administered via intravenous gtt at a dose of 200 mg every 3 weeks, Oxaliplatin at 130 mg/m^2^ every 3 weeks, and Tegafur at 40 mg twice daily for 2 weeks. A total of 3 cycles of neoadjuvant chemotherapy were completed.

Following the 3 cycle neoadjuvant chemotherapy, CT imaging suggested tumor downstaging, with a noticeable reduction in the size of the surrounding lymph nodes (Fig. 1D). Gastroscopy also showed a decrease in the size of the lesion, and endoscopic biopsy results indicated chronic inflammation (Fig. 1E and F). Based on these findings, tumor downstaging was confirmed. Subsequently in June 2020, the patient underwent a successful D2 radical gastrectomy (distal stomach, Fig. 1G), postoperative pathological examination revealed no residual tumor, confirming a pathological complete response (Fig. 1H). After surgery, the patient received 8 cycles of adjuvant therapy with the SOX regimen.

Follow-up CT scans conducted in 2022 and 2025 showed mild thickening of the residual stomach, without any abnormal enhancement. Since the initial diagnosis and subsequent neoadjuvant and surgical treatments, the patient has adhered to regular follow-up appointments. CT scans have not detected any signs of recurrence or metastasis. Circulating tumor cell (CTC) monitoring identified 2 mixed-type CTCs, with no interstitial CTCs detected. To date, the patient’s progression-free survival has reached 59 months.

## 3. Case presentation 2

**Figure 2.**
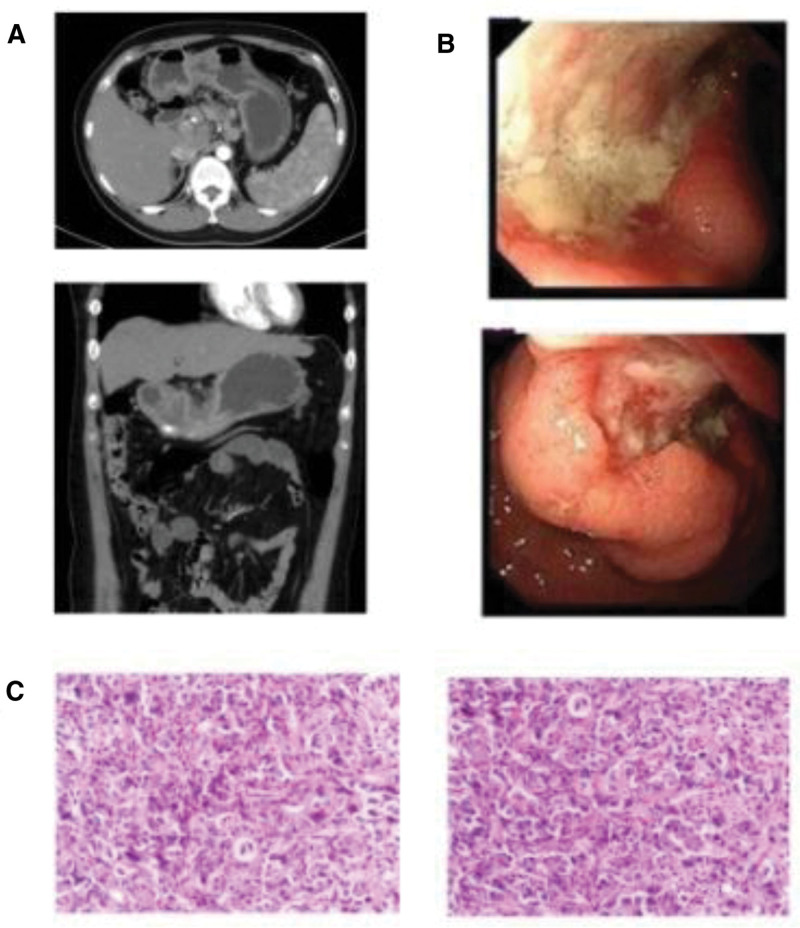
(A) Computed tomography before surgery; (B) gastroscopy showed advanced gastric cancer; (C) pathological examination.

In October 2023, a 37-year-old female patient presented to the hospital complaining of recurrent upper abdominal pain over the past 2 weeks. Hematological tests showed low levels of albumin (28.3 g/L) and prealbumin (62 mg/L). Tumor biomarker analysis revealed an elevated CA19-9 level of 41.02 ng/mL. CT scans showed discontinuous mucosa on the greater curvature of the gastric body near the antrum, while no abnormalities were observed in the peritoneal or retroperitoneal lymph nodes (Fig. 2A). Gastroscopy identified an advanced GC lesion measuring 2 × 3 cm at the gastric angle (Fig. 2B). Histopathological examination confirmed the diagnosis of adenocarcinoma (Fig. 2C). Based on endoscopic biopsy and imaging findings, the patient was diagnosed with gastric adenocarcinoma at stage cT3N1M0, or stage IIB.

According to the treatment guidelines for stage II non-esophagogastric junction tumors, radical gastrectomy is the recommended treatment. Considering the patient’s young age and our center’s experience indicating a poor prognosis for similar cases, neoadjuvant chemotherapy combined with surgical treatment was proposed. However, due to financial limitations, the patient declined neoadjuvant chemotherapy. As a result, a D2 radical gastrectomy (distal stomach) was performed. Postoperative pathological examination revealed a stage of pT3N3aMx, corresponding to stage IIIB. Immunohistochemical staining results were as follows: ERBR (‐), MLH1 (‐), MSH2 (+), MSH6 (+), PMS2 (‐), Her2 (1+), Ki67 (60% +), P53 (wild-type +), and PD-L1(+) with a CPS of 20. The details of the patient’s adjuvant therapy process remain unknown. Unfortunately, during the 1-year follow-up period, the patient succumbed to tumor recurrence.

## 4. Discussion

Two patients with advanced GC at the same stage exhibited markedly different prognoses due to varying treatment modalities. Although, theoretically, patients in stage IIIA have a poorer prognosis compared to those in stage IIB, neoadjuvant chemotherapy has the potential to significantly alter these outcomes. The preoperative staging assessment also seems to be less accurate for treatment selection.

We can make a hypothetical scenario: if patient of case 2 had received the same personalized treatment regimen as patient of case 1, or had been administered other neoadjuvant treatment regimens, would the prognosis of patient 2 have been drastically different? It is possible that tumor regression would have occurred, and the survival period might have been extended by 1 year, 2 years, or even longer. The distinct differences in the treatment directions chosen for these 2 cases have led to significant variations in their prognoses, prompting us to engage in in-depth reflection and inspiring new insights regarding the current treatment options for advanced GC. We have arrived at our perspective: neoadjuvant therapy should be recommended for advanced GC.

It is well known that the NCCN guidelines for the treatment of resectable advanced GC (cT2 or higher and any N) recommend surgery, neoadjuvant chemotherapy, or preoperative chemoradiotherapy. These guidelines also strongly emphasize that the optimal treatment option for any cancer patient is participation in clinical trials and actively encourage patients to enroll. The Japanese GC treatment guidelines indicate that neoadjuvant chemotherapy has become a standard treatment approach in Europe and the United States, with positive results reported from clinical trials conducted in China and North Korea. The Japanese guidelines suggest that cisplatin combined with S-1 as neoadjuvant therapy is highly effective and can be regarded as a standard treatment. However, it should be noted that the guidelines do not clearly specify a recommendation level for neoadjuvant chemotherapy in advanced GC, indicating that further validation is required in Japan. The CSCO treatment guidelines recommend that for stage II non-esophagogastric junction tumors, D2 radical gastrectomy plus adjuvant chemotherapy is an appropriate treatment option. For stage II esophagogastric junction tumors, neoadjuvant chemoradiotherapy followed by D2 radical gastrectomy is suggested. For stage III non-esophagogastric junction tumors, the choice between neoadjuvant chemotherapy and direct surgery remains a subject of controversy. For stage III esophagogastric junction tumors, neoadjuvant chemoradiotherapy and chemotherapy are recommended. Consequently, the necessity of neoadjuvant therapy for advanced GC remains a topic of ongoing debate. This manuscript will incorporate the neoadjuvant treatment regimen used in the case report to explore the development and current status of neoadjuvant chemotherapy for GC.

In 1982, Frei^[5]^ first introduced the concept of neoadjuvant chemotherapy (NACT) in oncology. NACT is defined as systemic chemotherapy administered before local treatment (surgery or radiotherapy) for malignant tumors, distinguishing it from postoperative adjuvant chemotherapy. After 40 years of development from concept to practical application, NACT has gradually established its significant value and its application scope has expanded to almost all malignant tumors. Both theoretical research and practical experience have demonstrated that NACT has 6 major advantages and has become an essential part of comprehensive cancer treatment^[6]^:

1. Neoadjuvant chemotherapy can initially target and kill tumor cells, reduce tumor size, alleviate tissue reactive edema, and decrease the invasion and adhesion between the tumor and surrounding tissues.
2. It can eliminate free tumor cells that have invaded blood vessels, thereby preventing hematogenous metastasis.
3. NACT can eradicate microscopic lymphatic metastases, reducing the risk of cancer recurrence.
4. It can inhibit the biological activity of free tumor cells, reducing the likelihood of their implantation and proliferation, and minimizing the risk of postoperative implantation and metastasis.
5. Neoadjuvant chemotherapy can achieve tumor down-staging, increasing the surgical resection rate.
6. By observing the tumor’s response to chemotherapy drugs during neoadjuvant treatment, it is possible to determine whether patients need to continue chemotherapy after surgery.

In 2005, the MAGIC study in the UK utilized the ECF regimen (Epirubicin + Cisplatin + Fluorouracil) for perioperative chemotherapy.^[7]^ In 2007, the FFCD9703 trial in France employed the CF regimen (Cisplatin + Fluorouracil). Patients treated with these 2 NACT regimens showed a significant improvement in 5-year survival rates, marking the beginning of NACT application in GC treatment. The A10-FLOT4 study in Germany in 2019 demonstrated that the perioperative FLOT regimen (Docetaxel + Oxaliplatin + Fluorouracil) had significantly better efficacy than the ECF regimen.^[8]^ NACT, represented by the FLOT regimen, has become the standard treatment for GC in Western countries and is widely used in clinical practice. However, it should be emphasized that the above-mentioned studies were all conducted on European and American populations. Due to ethnic differences, the application of the FLOT regimen in Asian populations is limited.

In China, the RESOLVE study,^[6]^ spearheaded by Peking University Cancer Hospital, and the RESONANCE study,^[9]^ led by the General Hospital of the Chinese People’s Liberation Army, have both demonstrated compelling evidence. Their findings indicate that NACT with the SOX regimen can significantly elevate the R0 resection rate, extend progression-free survival, and maintain acceptable levels of adverse reactions. Currently, the SOX regimen stands as the most widely adopted NACT regimen for GC in China. The initial patient we reported indeed underwent SOX NACT and achieved remarkable therapeutic outcomes. Nevertheless, a consensus regarding the selection of the optimal NACT regimen remains elusive. Moreover, the ideal timing for surgery and the most suitable number of neoadjuvant treatment cycles are still subjects of ongoing debate. The RESOLVE 2 study is designed to directly compare the efficacy and safety of the perioperative DOS regimen (Docetaxel + Oxaliplatin + Ticeo) with the SOX regimen. This endeavor aims to identify a novel adjuvant chemotherapy regimen with superior efficacy for patients with LAGC.

The encouraging efficacy of antiangiogenesis targeted drugs in the treatment of advanced GC has spurred researchers to explore their potential in the realm of neoadjuvant therapy. The results of the ST03 study revealed that the addition of bevacizumab to the ECX (Epirubicin + Cisplatin + Capecitabine) regimen during perioperative chemotherapy failed to confer survival benefits to patients with resectable esophagogastric cancer. In contrast, the phase II RAMSES/FLOT7 study demonstrated that incorporating ramucirumab into the perioperative treatment of FLOT could enhance the R0 resection rate.^[10]^ Another phase II clinical study^[11]^ suggested that the combination of Apatinib and the SOX regimen for the neoadjuvant treatment of LAGC exhibited significant efficacy with tolerable adverse reactions, achieving an R0 resection rate of 96.6%, a PCR rate of 13.8%, and a ypN0 rate of 39.3%. Although antiangiogenesis targeted drugs combined with chemotherapy have shown promising results in the neoadjuvant therapy of GC in previous studies, further large-scale investigations are essential to establish conclusive evidence.

Following the advancements in chemotherapy and targeted therapy, immunotherapy has emerged as a transformative force, poised to rewrite the overall cancer treatment paradigm. Its highly effective tumor debulking capabilities and substantial survival benefits have generated significant excitement.^[12]^ Previous research has indicated that the combination of immunotherapy and chemotherapy confers significant survival advantages in advanced unresectable gastric/esophagogastric junction tumors.^[13]^ Currently, several studies exploring the efficacy and safety of neoadjuvant immunotherapy in LAGC have been presented at major international conferences, such as the American Society of Clinical Oncology and the European Society for Medical Oncology. The 4 clinical studies of qNCT03939962, NCT03288350, NCT04354662, and NCT04341857 reported that the PCR rate of neoadjuvant immunotherapy for GC ranged from approximately 10% to 25%, while the R0 resection rate was around 92.6% to 98%. These findings underscore the promising application prospects of neoadjuvant immunotherapy for GC. The prospective randomized controlled trial, KEYNOTE 585, is designed to rigorously evaluate the efficacy and safety of pembrolizumab combined with chemotherapy in the neoadjuvant therapy of locally advanced gastric or gastroesophageal junction tumors. This study is expected to provide robust evidence supporting the role of neoadjuvant immunotherapy in GC treatment.

At the 2021 American Society of Clinical Oncology meeting, the results of a phase II clinical study exploring the combination of Camrelizumab, Apatinib, and SOX for neoadjuvant or conversion therapy in locally advanced (cT4a/b N+) GC were presented. The study enrolled a total of 25 patients, among whom 3 patients did not respond to the conversion therapy and 2 patients declined surgery. The final results showed a PCR rate of 16.7% and a tumor downstaging rate of 79.2% (19/24). A recent phase II clinical study in China further demonstrated that the combination of neoadjuvant immunotherapy, targeted therapy, and chemotherapy not only avoided increasing associated adverse effects but also significantly enhanced the efficacy of neoadjuvant therapy, with the tumor regression grade 1a/b reaching 33.3%.^[14]^ A phase II/III study (NCT04208347) is currently comparing the efficacy and safety of SOX, SOX plus Apatinib, and Camrelizumab in patients with HER2-negative LAGC. The interim results of G208 in 2024 strongly suggest that neoadjuvant targeted-immuno-chemotherapy can significantly improve the prognosis of patients with LAGC.

Collectively, a series of research results have illuminated a promising therapeutic landscape for neoadjuvant therapy of GC. When compared to the previous approach of simple surgical treatment, neoadjuvant therapy for GC appears to offer distinct advantages in terms of both safety and efficacy. Based on the clinical outcomes reported in our 2 cases, neoadjuvant therapy is likely to bring substantial benefits to patients with LAGC, regardless of the disease stage. We anticipate that future studies will continue to validate the merits of this therapeutic concept, and neoadjuvant therapy for locally advanced GC is poised to become the cornerstone of future GC treatment strategies.

*Limitations*: The lack of data on patient 2’s postoperative chemotherapy may make the article seem less rigorous; however, the value of data in such articles lies in its authentic presentation.

## Author contributions

**Data curation:** Xue Ding.

**Formal analysis:** Xue Ding, Li Dai, Hongbin Qi, Siqi Luo.

**Funding acquisition:** Xiangren Jin, Qian Wang.

**Resources:** Meifeng Zhang.

**Supervision:** Meifeng Zhang.

**Writing – original draft:** Meifeng Zhang.

**Writing – review & editing:** Xiangren Jin.
